# Genotyping by Sequencing and Genome–Environment Associations in Wild Common Bean Predict Widespread Divergent Adaptation to Drought

**DOI:** 10.3389/fpls.2018.00128

**Published:** 2018-02-21

**Authors:** Andrés J. Cortés, Matthew W. Blair

**Affiliations:** ^1^Department of Biological and Environmental Sciences, University of Gothenburg, Gothenburg, Sweden; ^2^Department of Agricultural and Environmental Science, Tennessee State University, Nashville, TN, United States

**Keywords:** drought tolerance, adaptation, genomic signatures of selection, divergent selection, SNP markers, Tajima’s D

## Abstract

Drought will reduce global crop production by >10% in 2050 substantially worsening global malnutrition. Breeding for resistance to drought will require accessing crop genetic diversity found in the wild accessions from the driest high stress ecosystems. Genome–environment associations (GEA) in crop wild relatives reveal natural adaptation, and therefore can be used to identify adaptive variation. We explored this approach in the food crop *Phaseolus vulgaris* L., characterizing 86 geo-referenced wild accessions using genotyping by sequencing (GBS) to discover single nucleotide polymorphisms (SNPs). The wild beans represented Mesoamerica, Guatemala, Colombia, Ecuador/Northern Peru and Andean groupings. We found high polymorphism with a total of 22,845 SNPs across the 86 accessions that confirmed genetic relationships for the groups. As a second objective, we quantified allelic associations with a bioclimatic-based drought index using 10 different statistical models that accounted for population structure. Based on the optimum model, 115 SNPs in 90 regions, widespread in all 11 common bean chromosomes, were associated with the bioclimatic-based drought index. A gene coding for an ankyrin repeat-containing protein and a phototropic-responsive NPH3 gene were identified as potential candidates. Genomic windows of 1 Mb containing associated SNPs had more positive Tajima’s D scores than windows without associated markers. This indicates that adaptation to drought, as estimated by bioclimatic variables, has been under natural divergent selection, suggesting that drought tolerance may be favorable under dry conditions but harmful in humid conditions. Our work exemplifies that genomic signatures of adaptation are useful for germplasm characterization, potentially enhancing future marker-assisted selection and crop improvement.

## Introduction

Understanding the genomic signatures associated with environmental variation is of great interest as it provides insights into how organisms adapt to their environment (Stinchcombe and Hoekstra, 2008; Hoffmann and Sgro, 2011; Savolainen et al., 2013). Recent genomic studies in wild plant populations have demonstrated that genome–environment associations (GEA) can be used to identify adaptive loci and predict phenotypic variation. Generally, the studies have associated single nucleotide polymorphism (SNP) alleles and parameters from the accessions’ environment of origin, to determine the potential for abiotic stress adaptation. For instance, Turner et al. (2010) predicted genetic adaptive variation to serpentine soils in *Arabidopsis lyrata*, Hancock et al. (2011) identified climate-adaptive genetic loci among a set of geographically diverse *Arabidopsis thaliana*, Fischer et al. (2013) predicted adaptive variation to topo-climatic factors in *Arabidopsis halleri*, Pluess et al. (2016) predicted genetic local adaptation to climate at a regional scale in *Fagus sylvatica*, and Yeaman et al. (2016) detected convergent local adaptation in two distantly related species of conifers.

The GEA approach has been also been explored in some crop accessions as a prospection strategy for discovering wild germplasm or landraces for breeding as an alternative to traditional phenotyping. For example, Lasky et al. (2012) and Des Marais et al. (2014) looked at natural variation in *Arabidopsis* for drought resistance and water use efficiency, Yoder et al. (2014) was able to capture adaptive variation to thermal tolerance, drought tolerance, and resistance to pathogens in *Medicago truncatula*, Lasky et al. (2015) predicted genotype × environment interactions to drought stress and aluminum toxicity in *Sorghum bicolor*, and Berthouly-Salazar et al. (2016) uncovered genomic regions involved in adaptation to abiotic and biotic stress on two climate gradients in *Cenchrus americanus*. Our group has focused on exploring the marker × environment association approach with wild relatives of the food crop common bean (*Phaseolus vulgaris* L.) and we have shown candidate genes such as DREB, ASR, and ERECTA to have haplotypes associated with drought tolerance (Cortés et al., 2012a,b; Blair et al., 2016). In this study, we couple genome–environment association with estimates of genome-wide diversity in wild accessions by using a whole-genome marker method with thousands of SNPs combined with climatic indices.

Among the most comprehensive marker systems for common bean SNP detection is the method called genotyping by sequencing (GBS). This technique has the flexibility of being adaptable to wild relatives with no previous sequence information needed although a reference genome for the species is useful (Elshire et al., 2011). To date, the GBS method has mostly been conducted for cultivars of common bean but rarely for wild accessions, which was one of the purposes of our research. A critical step for GBS assays is the preparation of restriction enzyme digested reduced representation libraries of genomic DNA that is barcoded for evaluation on a high throughput sequencer. As an initial example of the method for genetic mapping in common bean, Hart and Griffiths (2015) compared *Pst*I as a methylation sensitive enzyme to the non-methylation sensitive *Ape*K1 proposed for most small-genome species by Elshire et al. (2011) and evaluated a population of 84 lines and 12 parental checks. Bhakta et al. (2015) used the *PstI* GBS method to perform high resolution mapping on a population of 188 RILs from the cross of Jamapa × Calima, cultivated beans from opposite genepools and discovered nearly 2,000 usable SNP loci for genetic mapping. In parental surveys, Zou et al. (2013) used *Hae*III digestion and library construction in 36 Canadian small seeded breeding lines to discover nearly 25,000 physically tagged SNPs. Subsequently, Ariani et al. (2016) used another new enzyme, the four base pair cutter *Cvi*AII, to evaluate a broader range of 18 common beans (one ancestral wild, four Andean domesticates, four wild Mesoamericans and nine cultivated Mesoamerican) finding 3,200–21,000 SNPs/genotype. Schroder et al. (2016), in contrast, used a combination of *Mse*I and *Taq*I enzymes along with size selections to compare the feasibility of double digestion and small fragment size generation with four bp cutters, to the *Ape*K1 method for 25 Mesoamerican beans, finding up to 12.5 times more usable SNPs with an 8X coverage. More recently, Ariani et al. (2017) used the same protocol than Ariani et al. (2016) to reveal the spatial and temporal scales of range expansion in wild common bean. In all cases size selection, sequencing depth and a reference genome from Schmutz et al. (2014) were critical for calling SNPs and avoiding missing data or false positives due to poor sequence coverage. DARTseq, a modification of the GBS method, has been used on 188 cultivated Brazilian genotypes for diversity assessment finding its value in population structure analysis (Valdisser et al., 2017). With these results in the literature, we were confident that the GBS method would be practical for generating many SNPs in wild accessions of common bean and could be used for both diversity evaluation and association analysis.

Wild bean are thought to have diverged from its sister species in the tropical Andes (Rendón-Anaya et al., 2017) to later diversified in South and Central America from an original range in Central America, after which domestication in the southern and northern ends of each region gave origin to Andean and Mesoamerican domesticates, respectively (Gepts and Debouck, 1991; Kwak and Gepts, 2009; Schmutz et al., 2014). Studies of wild beans, show that despite the bimodality in domestication, they are loosely structured across the full range of environments from Northern Mexico to Northern Argentina, with identifiable sub-populations of wild types centered in Argentina/Southern Bolivia, Ecuador/Northern Peru, Colombia, Guatemala, Highland Mexico, and Lowland Mexico (Blair et al., 2012; Cortés, 2013; Cortés et al., 2013). The southernmost sub-population is typical of the Andean genepool while the two northern most sub-populations parallel the races found in Mesoamerican common bean with the centrally located sub-populations being intermediate. Among the wild beans many accessions survive and reproduce well in drought-affected regions of the New World, but have not been used for breeding the important seed types of the region.

Further wild germplasm characterization is important as common bean is a key source of nutrients and dietary protein for over 500 million people in Latin America and Africa and more than 4.5 out of 23 million hectares are grown in zones where drought is severe, such as in northeastern Brazil, coastal Peru, the central and northern highlands of Mexico, and in Eastern and Southern Africa (Broughton et al., 2003). This situation may become worst as increased drought due to climate change will reduce global crop production in >10% by 2050 with a potential to substantially worsen global malnutrition (Tai et al., 2014). Meanwhile wild beans are adapted as herbaceous species to dry forests and semi-arid regions of the Americas. Therefore, increasing drought tolerance in common bean commercial varieties is highly desirable and one potential source of resistance is from the wild accessions of the species. Given this, our milestones for this study were to: (1) implement GBS technology in wild accessions of common bean, (2) characterize geo-referenced climate information about the geographic origins of the wild accessions, and (3) find marker × environment associations at the whole genome level using the SNP loci discovered in the germplasm set. Specifically, we quantified SNP allelic associations with a bioclimatic-based drought index in order to identify adaptive variation suitable to breed drought tolerant varieties.

## Materials and Methods

### Plant Material and Compilation of the Bioclimatic-Based Drought Index

A total of 86 DNAs from *Phaseolus vulgaris* were used in this study, extracted from 86 geo-referenced wild common bean accessions (**Supplementary Table S1**). All the genotypes were provided by the Genetic Resources Unit at the International Center for Tropical Agriculture and are preserved under the treaty for genetic resources from the Food and Agriculture Organization hereafter abbreviated as the FAO collection. These accessions were selected to be a representative of a core collection for wild beans (Tohme et al., 1996) and all five subpopulations and genepools were uncovered by single-sequence repeat (SSR) markers by Blair et al. (2012). Geographic information provided for each accession by the Genetic Resource Unit^1^ was also used as described in Cortés et al. (2013) to estimate drought stress at each collection site based on the precipitation regimes coupled with potential evapotranspiration (PET) models (Thornthwaite and Mather, 1957; Hamon, 1961) accounting for the effects of historical temperature and radiation (Hijmans et al., 2005).

More concretely, monthly mean air temperature and monthly precipitation averaged for the years 1970–2000 were downloaded from WorldClim^2^ for each accession coordinate. The Thornthwaite method was then used to calculate PET (Cortés et al., 2013 for the detailed equations) considering the effects of both temperature and radiation (Thornthwaite and Mather, 1957). PET and the precipitation data were then combined in a monthly drought index that was averaged and normalized across all 12 months (Cortés et al., 2013 for the detailed equations). This normalized annual mean drought index (**Supplementary Figure S1**, Shapiro–Wilk normality test *P*-value = 0.106), fed by monthly environmental data averaged for the years 1970–2000, is hereafter referred as bioclimatic-based drought index. Two properties of this index must be noted. First, it assumes that plant distribution is in equilibrium with niche requirements and ecological forces, so that the errant presence of poorly adapted genotypes can be discarded (Forester et al., 2016). Second, this index is stable across years as it is based on climate data averaged across three decades. The ecological optimum, the stability and the normality of this index make it ideal for GEA analyses, in contrast with crude environmental measures.

### Sample Collection, DNA Extraction, and Genotyping-by-Sequencing

Leaf tissue weighing approximately 20 mg was harvested at 40 days after plant germination and was immediately dried in Silica Gel (Sigma–Aldrich, Germany). Genomic DNA was extracted using the QIAGEN DNeasy Plant Mini Kit (QIAGEN, Germany), following the manufacturer’s instructions, and quantified using a Qubit^®^ dsDNA HS Fluorometer (Life Technologies, Stockholm, Sweden). One 96-plex GBS assay was made according to Elshire et al. (2011) for the 86 accessions, with library preparation performed with *ApeKI* digestions at the Biotechnology Resource Center of the Institute for Genomic Diversity (Cornell University, United States). Genotyping and SNP calling were done with TASSEL software v. 3.0 (Glaubitz et al., 2014) based on the reference genome v. 2.0 for *P. vulgaris* deposited at the Phytozome website^3^. Sequence tags were aligned to the *P. vulgaris* reference genome (Schmutz et al., 2014), using the BWA method (Li and Durbin, 2007). A customized physical map was calculated and drawn using R v.3.3.1 (R Core Team) to place each new GBS locus.

### Identification of Loci Associated with the Bioclimatic-Based Drought Index

In order to account for possible demographic effects we examined subpopulation structure in the 86 geo-referenced wild common bean accessions using principal coordinates analysis (PCoA) implemented in the software Trait Analysis by aSSociation, Evolution and Linkage, Tassel v.5 (Bradbury et al., 2007). The same dataset and software were used to perform association analyses between the SNP markers and the bioclimatic-based drought index from Cortés et al. (2013). A total of ten generalized (GLM) and mixed (MLM) linear models were compared. Within each model family, five models were built as follows: (1) model with the genepool identity and the first two PCoA axes scores as covariates; (2) models with the within-genepool subpopulation identity, according to Blair et al. (2012), and the first two PCoA axes scores as covariates; (3) model with the first two PCoA axes scores as covariates; (4) model with the within-genepool subpopulation identity, according to Blair et al. (2012), as covariate; and (5) model with the genepool identity as covariate. Despite that GLMs usually exhibit a higher rate of false positives (Rosenberg et al., 2010), they were still considered in the present study for comparative purposes. All five MLMs used a centered IBS kinship matrix as a random effect to control for genomic background implementing the EMMA and P3D algorithms to reduce computing time (Zhang et al., 2010). QQ-plots of the *P*-values were inspected to assess whether excessive numbers of false positives were generated and choose in this way the optimum model. Significant associations were determined using a strict Bonferroni correction of *P*-values at alpha = 0.001, leading to a significance threshold of 4.4 × 10^-8^ (0.001 divided by the number of markers, 22,845) or -log_10_(4.4 × 10^-8^) = 7.36. The construction of customized PCoA and Manhattan diagrams was carried out with the software R v.3.3.1 (R Core Team).

Potential candidate genes were identified within the 1,000 bp sections flanking each SNP marker that was associated with the bioclimatic-based drought index by using the common bean reference genome (Schmutz et al., 2014) and the PhytoMine and BioMart tools in Phytozome v.12^3^.

### Sliding Window Analysis

We used a sliding window approach (window size = 1 × 10^6^ bps, step size = 200 kb) to describe patterns of variation and overall divergence across the genome. We computed per-window averages of SNP density, nucleotide diversity as measured by π (Nei, 1987), Watterson’s theta (𝜃) estimator (Watterson, 1975), and Tajima’s D (Tajima, 1989) using the software Tassel v.5 (Bradbury et al., 2007) and customized R scripts. Results of all windowed analyses were plotted against window midpoints in millions of base pairs (Mb) also using the software R v.3.3.1 (R Core Team).

Bootstrap-based means and 95% confidence intervals around the mean were calculated for these four summary statistics (SNP density, π, 𝜃 and Tajima’s D) when computed in sliding windows that contained or did not contain at least one marker that was associated with the bioclimatic-based drought index. Each summary statistic of windows containing and not containing associated SNPs was randomly resampled with replacement (bootstrapping) across windows within grouping factor (associated vs. no associated), and the overall mean was stored for each grouping factor. This step was iterated 1,000 times using customized R scripts. Bootstrapping was performed independently for each summary statistic in order to eliminate correlations among these.

## Results

### GBS Results

**FIGURE 1:**
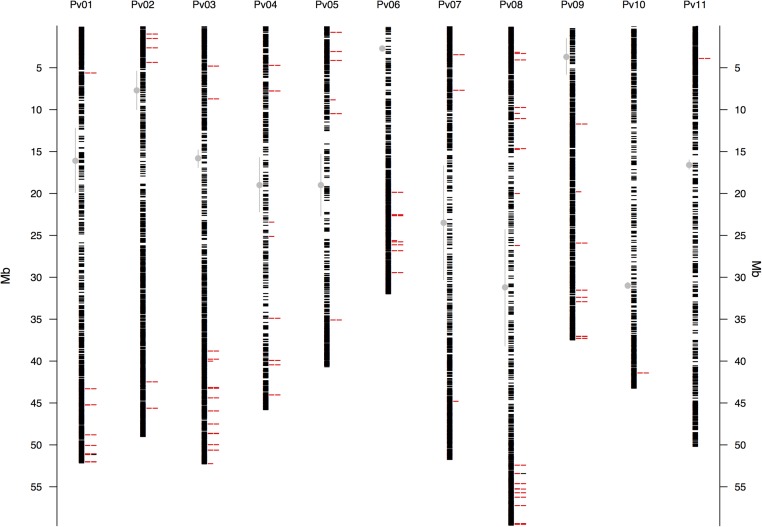
Physical map of the 22,845 GBS-derived SNP markers for all 11 common bean (Pv) chromosomes. Physical position is shown in millions of base pairs (Mb). Each black hyphen corresponds to a SNP marker and the first column of red hyphens indicate markers associated with the bioclimatic-based drought index (**Figure 3**), as calculated by Cortés et al. (2013). The second column of red hyphens marks the 75 associated regions that contained one gene (**Supplementary Table S3**). The two black hyphens in this column at chromosomes Pv1 and Pv8 indicate associated regions that had two genes. Regions are defined here as overlapping 1,000 bp sections that flanked associated markers. Vertical gray lines with a central filled gray dot mark the centromeres according to Schmutz et al. (2014).

The raw Illumina DNA sequence data (180,540,321 high-quality barcoded reads per lane) were processed through the GBS analysis pipeline as implemented in TASSEL-GBS v3.0. The GBS analysis generated 1,625,330 unique sequence clusters (tags, Elshire et al., 2011; Glaubitz et al., 2014). Of the total number of tags, 71.2% aligned uniquely to the *P. vulgaris* reference genome, 10.1% had multiple matches and 18.7% were unaligned. A total of 197,314 putative SNP markers where identified in the aligned tags. Of these, 47% with more than 20% missing data, a default threshold used for GBS studies (Glaubitz et al., 2014), and further 18% with minimum allele frequencies (MAF) below 0.05 were excluded from the dataset. The high number of missing data/unaligned sequences and low MAF value SNPs can be explained by the naturally high levels of sequence diversity in wild accessions (Glaubitz et al., 2014). Still the GBS project yielded 22,845 SNP markers of high quality with an average read depth of 13.6X gene coverage. The locations of these GBS-derived SNP loci for the wild accessions were identified by sequence similarity and drawn to scale based on their physical map distribution in the common bean genome (**Figure 1**). The centromeres according to Schmutz et al. (2014) were marked with the extent of the centromeric repeats in comparison with the GBS derived markers.

### There Were 115 Associated SNPs Widespread in 90 Regions in All 11 Chromosomes

**FIGURE 2:**
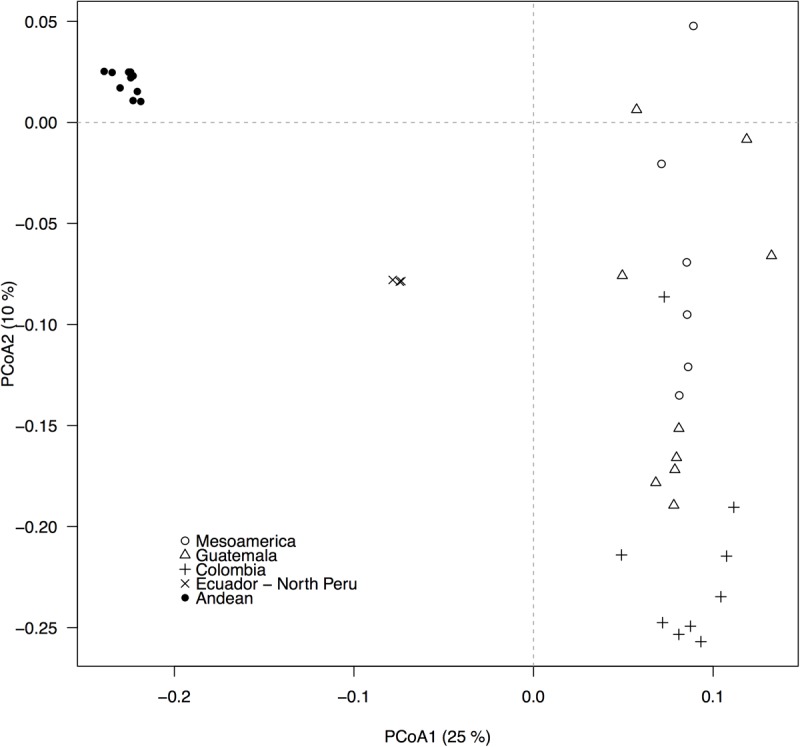
Population structure, as revealed by a principal coordinates analysis (PCoA), based on 22,845 GBS-derived SNP markers. Filled and empty symbols correspond to accessions from the Andean and the Mesoamerican genepool, respectively. Five within-genepool subpopulations, according to Blair et al. (2012), are indicated by different symbols. The percentage of explained variation by each axis is shown within parenthesis in the label of the correspond axis.

The GBS-derived SNP markers recovered the well-described Andean and Mesoamerican genepool structure and five within-genepool subpopulations, as depicted by the principal components analysis (**Figure 2**, compare to Blair et al., 2012). QQ-plots from the association analyses between the 22,845 SNP markers and the bioclimatic-based drought index from Cortés et al. (2013) indicated that GLM analyses likely had excessive rates of false positives (**Supplementary Figure S2**) whereas MLM models controlling for population structure and using a kinship matrix more effectively reduced the false positive rate (**Supplementary Figure S3**). The MLM model with the first two PCoA axes scores used as covariates, was the best at controlling for false positives because it exhibited the smoothest transition toward significant outliers.

**FIGURE 3:**
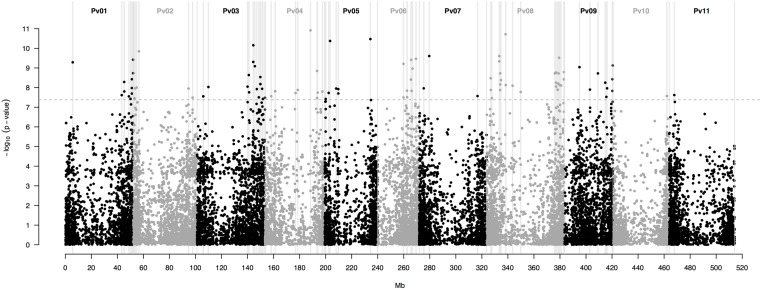
Manhattan plot of the optimum genome-environment association (GEA) analysis for drought tolerance in 86 common bean accessions based on 22,845 GBS-derived SNP markers. The bioclimatic-based drought index follows Cortés et al. (2013). The Manhattan plot despites per-marker –log_10_ (*P*-value) according to a mixed linear model with a centered IBS kinship matrix as a random effect, and the first two PCoA axes scores **(Figure 2)** as covariates. The gray dashed horizontal line marks the *P*-value threshold after Bonferroni-correction for multiple comparisons. Black and gray colors highlight different common bean (Pv) chromosomes. Gray vertical boxes indicate the 1,000 bp flanking region of each marker that was associated with the bioclimatic-based drought index.

This last model yielded a total of 115 SNP markers associated with the bioclimatic-based drought index at the Bonferroni-corrected significance threshold of 7.36 -log_10_ (*P*-value) (**Figure 3**). This group of SNPs had an average *R*^2^ of 51.3% ± 0.4, fairly stable across markers (**Supplementary Table S2**). The 115 SNPs were clustered in 90 different regions, defined here as overlapping 1,000 bp sections that flanked associated markers. Associated SNPs and regions were widespread in all 11 common bean chromosomes (**Table 1**).

**Table 1:** Per-chromosome (Pv) summary statistics for the 115 SNP markers associated with the bioclimatic-based drought index (Cortés et al., 2013) in 86 common bean accessions based on the optimum association analysis (**Figure 3**).

Pv	Number of associated SNPs	Average –log_10_ (*P*-value)	Average *R*^2^ (%)	Number of associated regions	Number of regions with more than one associated SNP	Average number of associated SNPs in regions with more than one associated SNP	Number of associated regions containing genes	Number of genes
Pv1	11	8 ± 2	51 ± 1	11	0	NA	10	11
Pv2	6	8 ± 3	50 ± 2	6	0	NA	6	6
Pv3	21	8 ± 2	51 ± 1	16	4	2.25	14	14
Pv4	11	8 ± 2	50 ± 1	8	2	2.50	6	6
Pv5	7	8 ± 3	52 ± 2	6	1	2.00	5	5
Pv6	12	8 ± 2	52 ± 1	8	4	2.00	7	7
Pv7	3	8 ± 5	50 ± 2	3	0	NA	2	2
Pv8	32	8 ± 1	52 ± 1	21	5	3.20	15	16
Pv9	9	8 ± 3	51 ± 1	9	0	NA	8	8
Pv10	2	7.6	47.8	1	1	2.00	1	1
Pv11	1	7.6	47.1	1	0	NA	1	1
Total	115	8 ± 1	51.3 ± 0.4	90	17	2.47	75	77

*Given are results of a mixed linear model with a centered IBS kinship matrix as a random effect, and the first two PCoA axes scores (**Figure 2**) as covariates. Regions are defined here as overlapping 1,000 bp sections that flanked associated markers. Potential candidate genes were identified within the 1,000 bp section flanking each associated marker.*

Chromosomes Pv3 and Pv8 had the highest number of associated SNPs with 21 and 32 SNPs clustered in 16 and 21 different regions, respectively. Chromosomes Pv1, Pv2, Pv4, Pv5, Pv6, and Pv9 contained an intermediate number of associated SNPs with 11, 6, 11, 7, 12, and 9 SNPs clustered in 11, 6, 8, 6, 8, and 9 different regions, respectively. Chromosomes Pv7, Pv10 and Pv11 had the fewest number of associated SNPs with 3, 2, and 1 SNPs clustered in 3, 1, and 1 different regions, respectively. Chromosome Pv8 had more regions with at least two associated SNPs than any other chromosome, and these regions had more associated SNPs that in any other chromosome for a total of five regions with an average number of 3.2 associated SNPs in each region. The single region that contained the most associated SNPs was also situated on chromosome Pv8 with 6 SNPs and an average *R*^2^ of 51.1% ± 0.3. After this chromosome, Pv3 was also notable for having four regions with an average number of 2.5 associated SNPs per region.

A total of 75 regions, comprising 99 SNP markers associated with the bioclimatic-based drought index, contained at least one gene, for a total of 77 genes identified as potential candidates for drought tolerance from the wild accession analysis (**Supplementary Table S3**). Most genes were from chromosomes Pv1, Pv3, and Pv8 with 11, 14, and 16 genes. Only two regions, at chromosomes Pv1 and Pv8 and containing a total of seven different SNPs, spanned two or more genes. The one in Pv8 was the region with more associated SNPs. One of the two genes in this region encoded an ankyrin repeat-containing protein, which was associated with osmotic regulation via the assembly of cation channels in the membranes (Voronin and Kiseleva, 2008). Among other identified potential candidate genes there was a phototropic-responsive NPH3 gene (Pedmale and Liscum, 2007) in Pv3. The associated SNPs flanking these genes had an average *R*^2^ of 49% ± 0.2.

### Associated Genomic Windows Were Enriched for SNP Density and Positive Tajima’s D Scores

**FIGURE 4:**
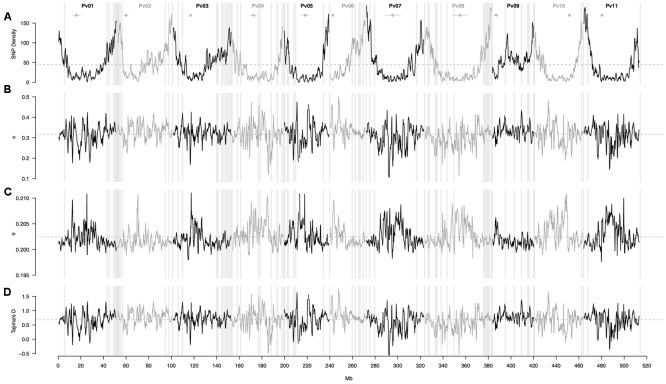
Patterns of genome-wide diversity in 86 common bean accessions based on 22,845 GBS-derived SNP markers. A sliding window analysis (window size = 1 × 10^6^ bp, step size = 200 kb) was used to compute **(A)** SNP density, **(B)** nucleotide diversity as measured by π, **(C)** Watterson’s theta estimator (𝜃), and **(D)** Tajima’s D. Results of all windowed analyses are plotted against window midpoints in millions of base pairs (Mb). Black and gray colors highlight different common bean (Pv) chromosomes. Gray dashed horizontal lines indicate genome-wide averages. Gray vertical boxes indicate the 1,000 bp flanking region of each marker that was associated with the bioclimatic-based drought index **(Figure 3)**. Horizontal gray lines with a central filled gray dot at the top of the figure mark the centromeres according to Schmutz et al. (2014).

A sliding window analysis (window size = 1 × 10^6^ bp, step size = 200 kb) was used to explore the patterns of genome-wide diversity. Marker density decayed drastically toward the centromeres. Average marker density was 44 SNPs per million base pairs (95% CI, 4–143, **Figure 4A**). Average nucleotide diversity as measured by π was 0.3 per million base pairs (95% CI, 0.2–0.4, **Figure 4B**). Average Watterson’s theta (𝜃) was 0.20 per million base pairs (95% CI, 0.19–0.21, **Figure 4C**). Average Tajima’s D was 0.68 per million base pairs (95% CI, 0.05–1.22, **Figure 4D**).

**FIGURE 5:**
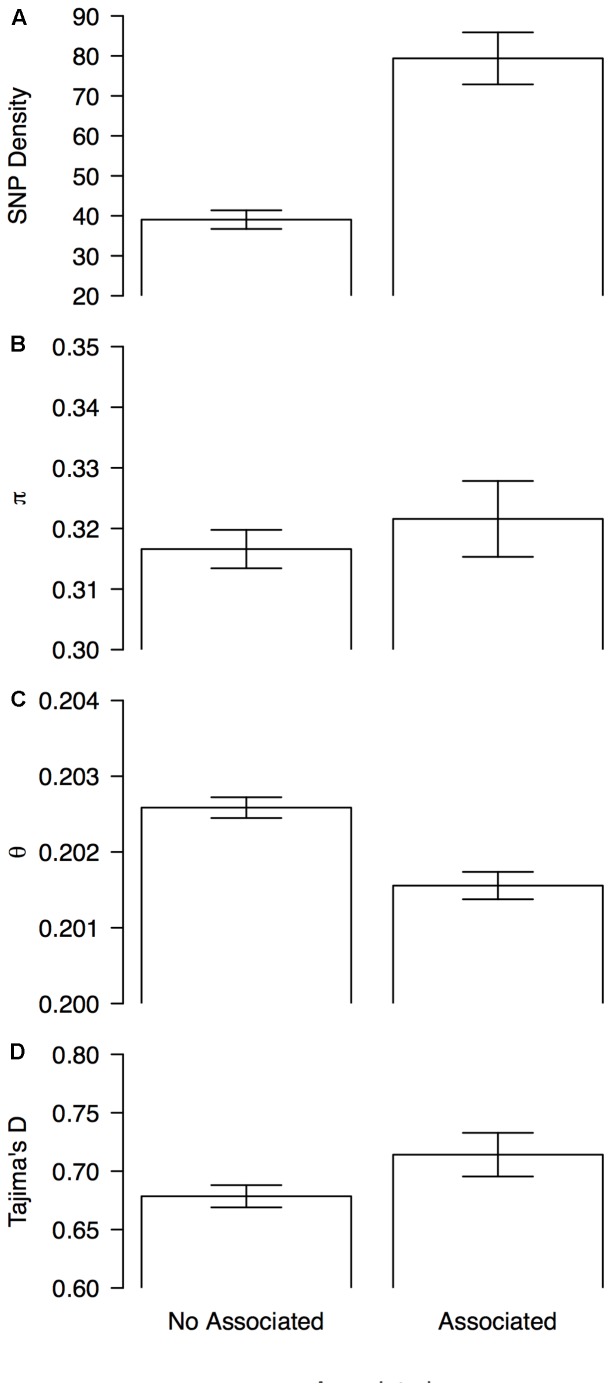
Patterns of genome-wide diversity in genomic windows associated and no associated with drought tolerance. Means and 95% permutation-based confidence intervals are shown for **(A)** SNP density, **(B)** nucleotide diversity as measured by π, **(C)** Watterson’s theta estimator (𝜃), and **(D)** Tajima’s D, when computed in sliding windows (window size = 1 × 10^6^ bp, step size = 200 kb) that contained (associated) or did not contained (no associated) at least one marker that was associated with the bioclimatic-based drought index (**Figure 4**).

These statistics were compared between 1 Mb sliding windows that contained (associated windows) or did not contain at least one marker that was associated with the bioclimatic-based drought index (non-associated windows). Genomic windows containing at least one associated SNP had overall higher SNP density (79 ± 6 vs. 39 ± 2, **Figure 5A**), lower scores for Watterson’s theta (𝜃) scores (0.2016 ± 0.0001 vs. 0.2026 ± 0001, **Figure 5C**) and more positive Tajima’s D scores (0.71 ± 0.02 vs. 0.678 ± 0.009, **Figure 5D**) than windows without associated markers. Nucleotide diversity, as measured by π, was slightly elevated in associated windows when compared with no associated windows (0.322 ± 0.006 vs. 0.317 ± 0.003, **Figure 5B**).

## Discussion

This research indicates that adaptation to drought in common bean, as estimated by bioclimatic variables, is widespread across all 11 common bean chromosomes and has been under natural divergent selection (inflated Tajima’s D values). Both of these results imply that the genetic sources for drought tolerance are abundant in wild common bean and that natural drought tolerance may be favorable under dry conditions but harmful in more humid conditions. The awareness about this trade-off, upon which natural divergent selection has been acting for thousands of years, may aid the breeding of new drought tolerant varieties specifically adapted to unique micro-environments and local regions rather than varieties, eventually obsolete, originally intended for a wider range of environments. Below we first discuss the evidence that support the main conclusion of widespread divergent adaptation to drought. Later, we follow up by discussing the implications of this finding.

### Wild Common Bean Exhibit Widespread Divergent Adaptation to Drought

It is well known that selective processes, such as purifying selection and local adaptation (divergent selection), differentially imprint regions within the same genome, causing a heterogeneous departure of genetic variation from the neutral expectations and from the background trend (Ellegren and Galtier, 2016). Specifically, habitat-mediated purifying selection is associated with localized low values of nucleotide diversity (π) (Nei, 1987) and Tajima’s D (Tajima, 1989) and high scores of the Watterson’s theta (𝜃) estimator (Watterson, 1975) because only low-frequency polymorphisms can avoid being eliminated by widespread directional selection. Although recent population bottlenecks tend to achieve the same reduction in nucleotide variation, this pattern is expected at a more genome-wide level. Similarly, local adaptation tends to homogenize haplotypes within the same niche and fix polymorphisms in different populations. Consequently, few haplotypes with high frequency are retained, corresponding to high values of nucleotide diversity (π) and Tajima’s D, and low scores of the Watterson’s theta (𝜃) estimator (Wakeley, 2008). While independent domestication events, extensive population structure, and population expansions after bottlenecks can produce the same patterns (Nei, 2010), these demographic processes are expected to be observed at a more genome-wide level.

Since divergent selection tends to homogenize haplotypes within the same niche and fix polymorphisms in different populations, few haplotypes with high frequency are retained, matching high values of nucleotide diversity and Tajima’s D, and low values of the Watterson’s theta (𝜃) estimator (Wakeley, 2008). In this study we have found that the genomic regions associated with the bioclimatic-based drought index, widespread in all 11 common bean chromosomes, displayed these very same signatures: inflated Tajima’s D values and lowered Watterson’s theta (𝜃) estimator. In other words, few haplotypes with high frequency are being differentially fixed in populations coming from contrasting environments. This result speaks for divergent selection acting on the genetic variants that are associated with drought tolerance, as compared to the opposite signal of directional selection, in which few dominant haplotypes are expected.

Yet, genomic signatures associated with habitat heterogeneity can still result from causes other than adaptation and selection (Nei, 2010), for example random genetic drift and population structure, and are also influenced by differences in ancestral variation and recombination in the genome (Strasburg et al., 2011; Cortés, 2015; Cortés et al., 2015a; Ellegren and Galtier, 2016; Wolf and Ellegren, 2016). Moreover, the origin of habitat-associated variants from novel or standing genetic variation leads to distinctively different patterns of genomic divergence (Hermisson and Pennings, 2005; Barrett and Schluter, 2008; Pritchard et al., 2010). A way to distinguish these underlying causes of divergence is comparing summary statistics (i.e., Tajima’s D) from different genomic sections, as we did here, because demographic processes usually leave genome-wide signatures while selection tends to imprint more localized regions (Wakeley, 2008). With this in mind, the signatures of divergent selection displayed in the genomic regions that in this study were associated with the bioclimatic-based drought index are unlikely due to confounding demographic processes such as independent domestication events, extensive population structure and population expansions after bottlenecks because the mixed linear model that we chose to identify the GEA accounted for population structure and demographic processes usually leave genome-wide signatures that should have imprinted the no associated windows as well. Therefore, the signatures displayed on the genetic variants that are associated with drought tolerance seem to reflect true divergent selection rather that confounding demographic processes like independent domestication events, extensive population structure, or population expansions after bottlenecks.

One potential caveat may concern the fact that we used GBS with the *ApeK1* restriction enzyme for the first time in wild accessions of common bean, possibly leading to problems like missing data due to sequence divergence compared to the cultivated reference genome. Nonetheless, wild-cultivated divergent regions with a high proportion of missing data seem to be rare because the U-shaped pattern of SNP density is just as expected when using a non-methylation sensitive enzyme. This decay in diversity proportional to the decay in the rate of recombination (toward the centromeres) was first described in *D. melanogaster* and has been confirmed in many organisms since then (Ellegren and Galtier, 2016). The pattern was understood as an effect of genetic hitchhiking, but background selection may also be a contributing factor, perhaps even the dominating one.

It is also appropriate to clarify that the power to detect marker-trait associations in this study was not limited by the number of markers. Every three SNPs are redundant and replaceable given a criterion of linkage disequilibrium, or LD (Carlson et al., 2004), which is higher in selfing plants (Slatkin, 2008; Rossi et al., 2009), like common bean (Broughton et al., 2003; Rossi et al., 2009; Blair et al., 2018). At least in humans, a 0.8 *R*^2^ threshold is used to identify maximally informative SNPs for association analyses by reducing the redundancy due to LD (Carlson et al., 2004), and this could apply to other species as well (Slate et al., 2009). Here, average marker density was 44 SNPs per million base pairs, or average distance between any two SNP markers in the reference genome was 23 thousand base pairs. Since LD in wild common bean, measured as marker correlation *R*^2^, was reported to decay to 0.8 per every 81 thousand base pairs (Rossi et al., 2009; Blair et al., 2018), on average every 3, physically linked, GBS-derived SNP markers are in sufficient LD to be considered redundant by the association analysis.

Finally, despite that the modest sample size used in the association analysis may overlook the majority of associations with low effect sizes and large-effect genes that segregate at low frequency (Maher, 2008), genes with major effects that segregate at moderate frequencies are still identifiable. In fact, this may be the case of a gene coding for an ankyrin repeat-containing protein, associated with osmotic regulation (Voronin and Kiseleva, 2008), and a phototropic-responsive NPH3 gene (Pedmale and Liscum, 2007), found in regions in chromosomes Pv8 and Pv3 flanking associated SNPs with an average *R*^2^ of 49%.

### Wild Accessions May Be Useful to Breed Drought Tolerant Varieties in Common Bean

Previous research from Tiranti and Negri (2007) and Ariani et al. (2017) demonstrated that selective micro-environmental effects play a role in shaping genetic diversity and structure in common bean wild accessions. In this study, we have confirmed that ecological gradients related with drought stress are associated with divergent selection in wild common beans, after accounting for genepool and subpopulation structure. This divergent selective pressure might be a consequence of local level rainfall patterns. Specifically, in tropical environments near the equator with bimodal rainfall a mid-season dry period occurs that can last 2–4 weeks. In contrast in the sub-tropics, a dry period of three or more months can occur. In response to this mid-cycle drought of the sub-tropics, *P. vulgaris* enters a survival mode of slow growth and reduced physiological activity until rainfall resumes and flowering occurs (Beebe et al., 2008). Beans growing in wetter conditions on the other hand are less frequently subjected to these environmental pressures and have a fitness advantage to mature in a shorter length of time. Given these ecological differences, the reaction typically associated with drought tolerance although favorable under dry conditions seems detrimental under more humid conditions, which is consistent with the genomic signature of divergent selection observed in this study. This trade-off must be accounted for when breeding for drought tolerance, reinforcing the need of varieties locally adapted to unique micro-environments and narrow regions instead of varieties intended for a wider range of environments (Cortés and Blair, 2018a,b).

Furthermore, wild accessions of common bean occupy more geographical regions with extensive drought stress than cultivated accessions (Cortés et al., 2013). Those regions include the arid areas of Peru, Bolivia, and Argentina, and the valleys of northwest Mexico. Hence, a broad habitat distribution for wild common bean has exposed these genotypes to both dry and wetter conditions, while cultivated common bean has a narrower distribution and is traditionally considered susceptible to drought. These differences in the ecologies of wild and cultivated common bean have been associated with higher genetic diversity in the former group when surveying candidate genes for drought tolerance such as the ASR (Cortés et al., 2012a), DREB (Cortés et al., 2012b), and ERECTA (Blair et al., 2016) gene families, once population structure (Blair et al., 2012) and the background distribution of genetic diversity have been accounted for.

Although not all associated markers may be causal, but rather linked with causal elements, in this study we were able to identify a wide genetic source for drought tolerance, including new potential candidates like a gene coding for an ankyrin repeat-containing protein (Voronin and Kiseleva, 2008) and a phototropic-responsive NPH3 gene (Pedmale and Liscum, 2007). We were also able to identify some further differences between the adaptations of wild accessions found in arid and more humid environments, which may be valuable for plant breeding. Therefore, we reinforce, as was envisioned by Acosta et al. (2007), that wild accessions and landraces of common bean be taken into account to exploit naturally available divergent variation for drought tolerance.

## Perspectives

The potential candidate genes identified in this study should be validated as candidates for drought tolerance through re-sequencing and controlled drought stress treatments under green house and field conditions, ideally in a diverse panel of common bean accessions and closely related species, such as Tepary bean (*P. acutifolius*) that is known for growing in desert and semi-arid environments. Our group is currently running this initiative as part of a larger project spanning several other candidate genes, such as DREB, ASR, and ERECTA (Cortés et al., 2012a,b; Blair et al., 2016).

The work presented here ultimately illustrates that genomic signatures of adaptation are useful for germplasm characterization, potentially enhancing future marker-assisted selection and crop improvement. We envision that GEA studies coupled with estimates of genome-wide diversity will become more common in the oncoming years. They both might allow assessing the naturally available genetic variability for adaptation to various types of stress that, like drought, are expected to worsen with climate change. For instance, frost (Wheeler et al., 2014, 2016), nutrient limitation (Sedlacek et al., 2014; Little et al., 2016), altered snowmelt (Cortés et al., 2015b; Cortés, 2016, 2017a), distorted biotic interactions (Wheeler et al., 2015), and altered growing (Sedlacek et al., 2015, 2016) and flowering (Cortés et al., 2014) seasons, can easily be modeled and associated with allelic variants using a genome-wide diversity background. GEA studies coupled with estimates of genome-wide diversity could also be expandable to heterogeneous collections that include a mix of commercial accessions, landraces, wild populations and wild relatives, and that span a variety of ecologically diverse managed and natural ecosystems (Madriñán et al., 2013; Cortés and Wheeler, 2018) screened with a wide range of genotyping techniques (Cortés et al., 2011; Galeano et al., 2012; Kelleher et al., 2012; Blair et al., 2013). Genomic selection models, which are becoming increasingly popular (Desta and Ortiz, 2014), in conjunction with a wide-spectrum of stochastic models (Cortés, 2017b, 2018), could even incorporate at some point environmental variables and estimates of genome-wide diversity in order to improve the prediction of phenotypic variance and the estimation of the genotype × environment interactions.

## Data Accessibility

The pipeline configuration file and SNP dataset are archived at the Dryad Digital Repository under doi: 10.5061/dryad.j2c24.

## Author Contributions

AC designed the study with insights from MB. AC collected and analyzed the data. AC wrote the manuscript with contributions from MB.

## Conflict of Interest Statement

The authors declare that the research was conducted in the absence of any commercial or financial relationships that could be construed as a potential conflict of interest.

## Footnotes

1 genebank.ciat.cgiar.org

2 http://www.worldclim.org

3 https://phytozome.jgi.doe.gov/pz/portal.html

## Supplementary Material

FIGURE S1 Boxplot and histogram of the bioclimatic-based drought index (DI), according to Cortés et al. (2013), for the 86 common bean accessions used in this study.

FIGURE S2 Genome–environment association (GEA) analyses for drought tolerance according to five different general linear models (GLMs) in 86 common bean accessions based on 22,845 GBS-derived SNP markers. Given are Manhattan plots **(A**,**C**,**E**,**G**,**I)** and QQ-plots **(B**,**D**,**F**,**H**,**J)** of -log_10_ (*P*-value) for the following models: **(A**,**B)** GLM with the genepool identity and the first two PCoA axes scores **(****Figure 2****)** as covariates; **(C**,**D)** GLM with the within-genepool subpopulation identity, according to Blair et al. (2012), and the first two PCoA axes scores **(****Figure 2****)** as covariates; **(E**,**F)** GLM with the first two PCoA axes scores **(****Figure 2****)** as covariates; **(G**,**H)** GLM with the within-genepool subpopulation identity, according to Blair et al. (2012), as covariate; and **(I**,**J)** GLM with the genepool identity as covariate. The bioclimatic-based drought index follows Cortés et al. (2013). The gray dashed horizontal line marks the *P*-value threshold after Bonferroni-correction for multiple comparisons. Black and gray colors highlight different common bean (Pv) chromosomes.

FIGURE S3 Genome–environment association (GEA) analyses for drought tolerance according to five different mixed linear models (MLMs) in 86 common bean accessions based on 22,845 GBS-derived SNP markers. Given are Manhattan plots **(A**,**C**,**E**,**G**,**I)** and QQ-plots **(B**,**D**,**F**,**H**,**J)** of –log_10_ (*P*-value) for the following models: **(A**,**B)** MLM with the genepool identity and the first two PCoA axes scores **(****Figure 2****)** as covariates; **(C**,**D)** MLM with the within-genepool subpopulation identity, according to Blair et al. (2012), and the first two PCoA axes scores **(****Figure 2****)** as covariates; **(E**,**F)** MLM with the first two PCoA axes scores **(****Figure 2****)** as covariates (model shown in **Figure 3**); **(G**,**H)** MLM with the within-genepool subpopulation identity, according to Blair et al. (2012), as covariate; and **(I**,**J)** MLM with the genepool identity as covariate. All models used a centered IBS kinship matrix as a random effect. The bioclimatic-based drought index follows Cortés et al. (2013). The gray dashed horizontal line marks the *P*-value threshold after Bonferroni-correction for multiple comparisons. Black and gray colors highlight different common bean (Pv) chromosomes.

TABLE S1 Identity of the 86 common bean accessions used in this study. The G identification number (from the Genetic Resources Unit at the International Center for Tropical Agriculture) and the country of origin are shown according to the following convention: ARG for Argentina, BOL for Bolivia, COL for Colombia, ECU for Ecuador, GTM for Guatemala, HND for Honduras, MEX for Mexico and PER for Peru. The bioclimatic-based drought index (DI) according to Cortés et al. (2013) is also shown (and in **Supplementary Figure S1**). It appears in red if the accession was found in a dry environment (high drought index, above zero) or in blue if it was found in a very wet habitat (low drought index, below zero).

TABLE S2 Statistics from the optimum association analysis for the 115 SNP markers associated with the bioclimatic-based drought index in 86 common bean accessions based on 22,845 GBS-derived SNP markers. Given are results of a mixed linear model (depicted in **Figure 3**) with a centered IBS kinship matrix as a random effect, and the first two PCoA axes scores (**Figure 2**) as covariates. Among other statistics, the *F*-value, the -log_10_ (*P*-value) and the variation explained (*R*^2^) by each marker, are shown. Marker names are given as chromosome and position on the common bean reference genome from Schmutz et al. (2014). Unique regions containing one or more SNPs are named with a consecutive number per chromosome under the column called “region.” Gray cells in this column mark regions with two or more associated SNPs. Regions are defined here as overlapping 1,000 bp sections that flanked associated markers.

TABLE S3 List of 77 genes in the 75 associated regions that contained at least one gene. The position of each gene and its functional description are shown together with other identifiers and descriptors. The list of associated SNP markers surrounding each gene is also depicted, for a total of 99 SNPs. Genes co-occurring in the same region are marked with gray cell under the column “associated SNPs.” Regions are defined here as overlapping 1,000 bp sections that flanked associated markers.
