# Supplementary figures and images for: Genotyping by Sequencing and Genome–Environment Associations in Wild Common Bean Predict Widespread Divergent Adaptation to Drought
Source: Front Plant Sci. 2018 Feb 21;9:128. doi: 10.3389/fpls.2018.00128 (PMC5826387; doi:10.3389/fpls.2018.00128)

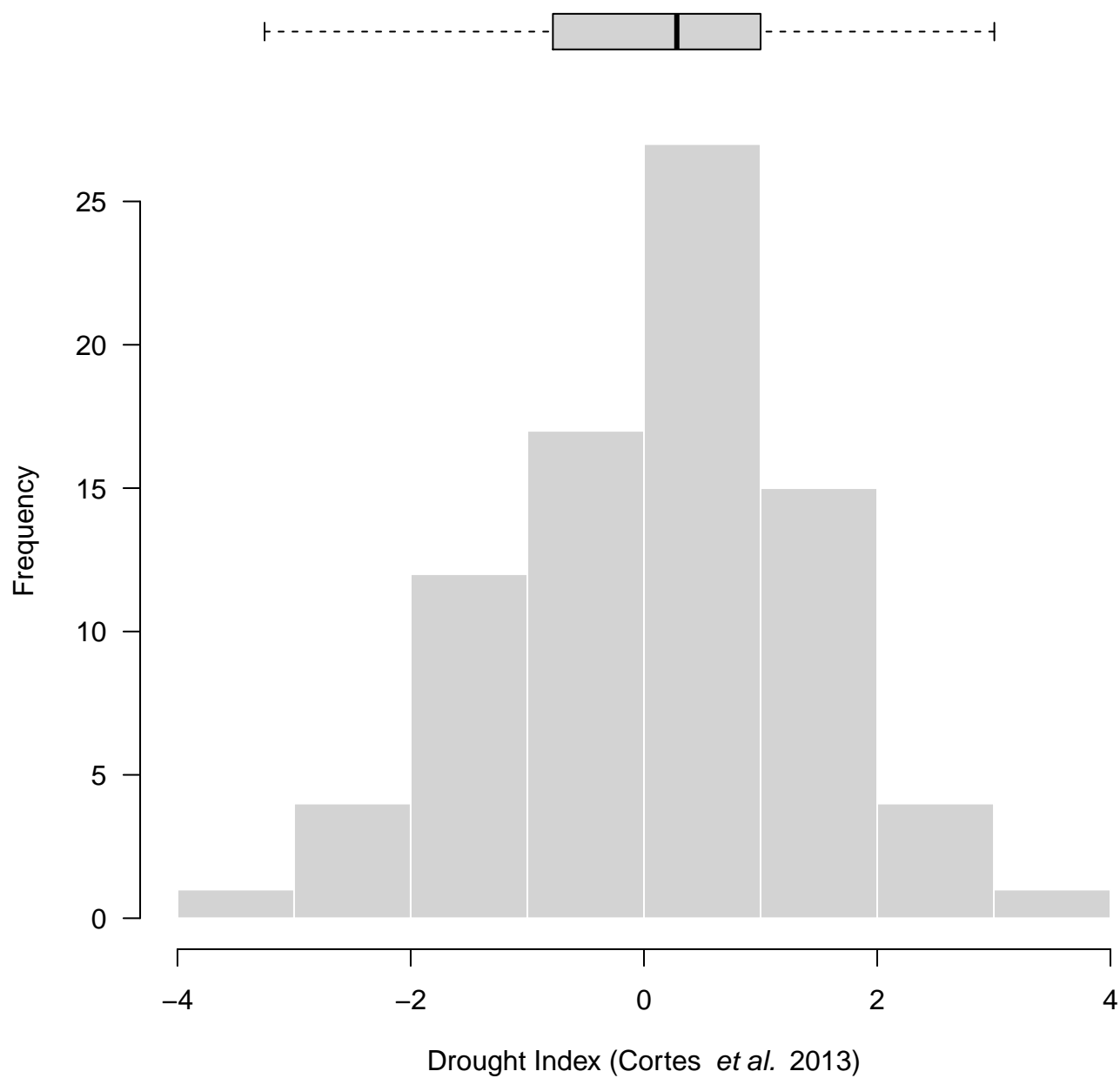

Supplement: FIGURE S1 — Boxplot and histogram of the bioclimatic-based drought index (DI), according to Cortés et al. (2013), for the 86 common bean accessions used in this study. [file Image_1.pdf]

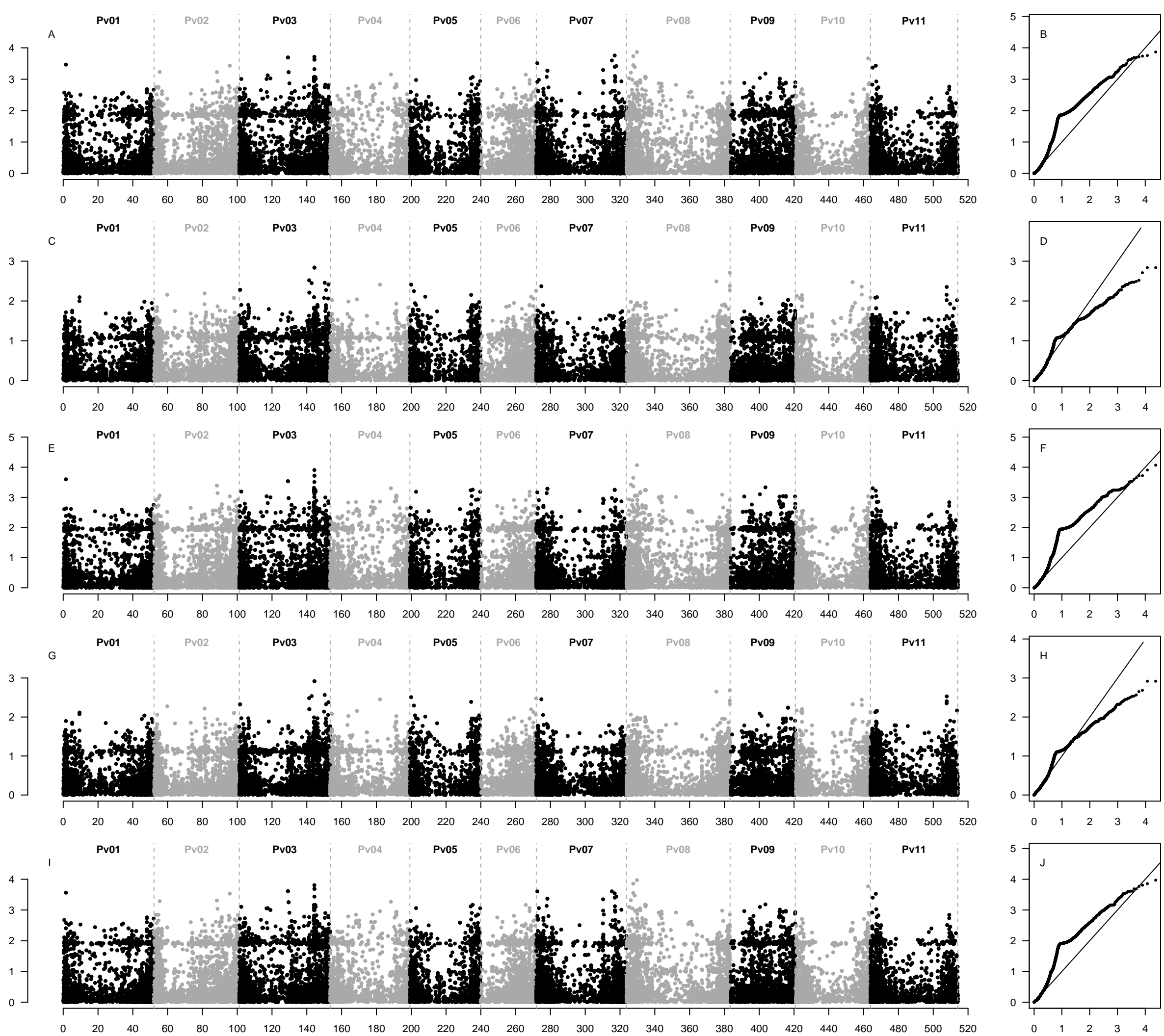

Supplement: FIGURE S2 — Genome–environment association (GEA) analyses for drought tolerance according to five different general linear models (GLMs) in 86 common bean accessions based on 22,845 GBS-derived SNP markers. Given are Manhattan plots (A,C,E,G,I) and QQ-plots (B,D,F,H,J) of -log10 (P-value) for the following models: (A,B) GLM with the genepool identity and the first two PCoA axes scores (Figure 2) as covariates; (C,D) GLM with the within-genepool subpopulation identity, according to Blair et al. (2012), and the first two PCoA axes scores (Figure 2) as covariates; (E,F) GLM with the first two PCoA axes scores (Figure 2) as covariates; (G,H) GLM with the within-genepool subpopulation identity, according to Blair et al. (2012), as covariate; and (I,J) GLM with the genepool identity as covariate. The bioclimatic-based drought index follows Cortés et al. (2013). The gray dashed horizontal line marks the P-value threshold after Bonferroni-correction for multiple comparisons. Black and gray colors highlight different common bean (Pv) chromosomes. [file Image_2.pdf]

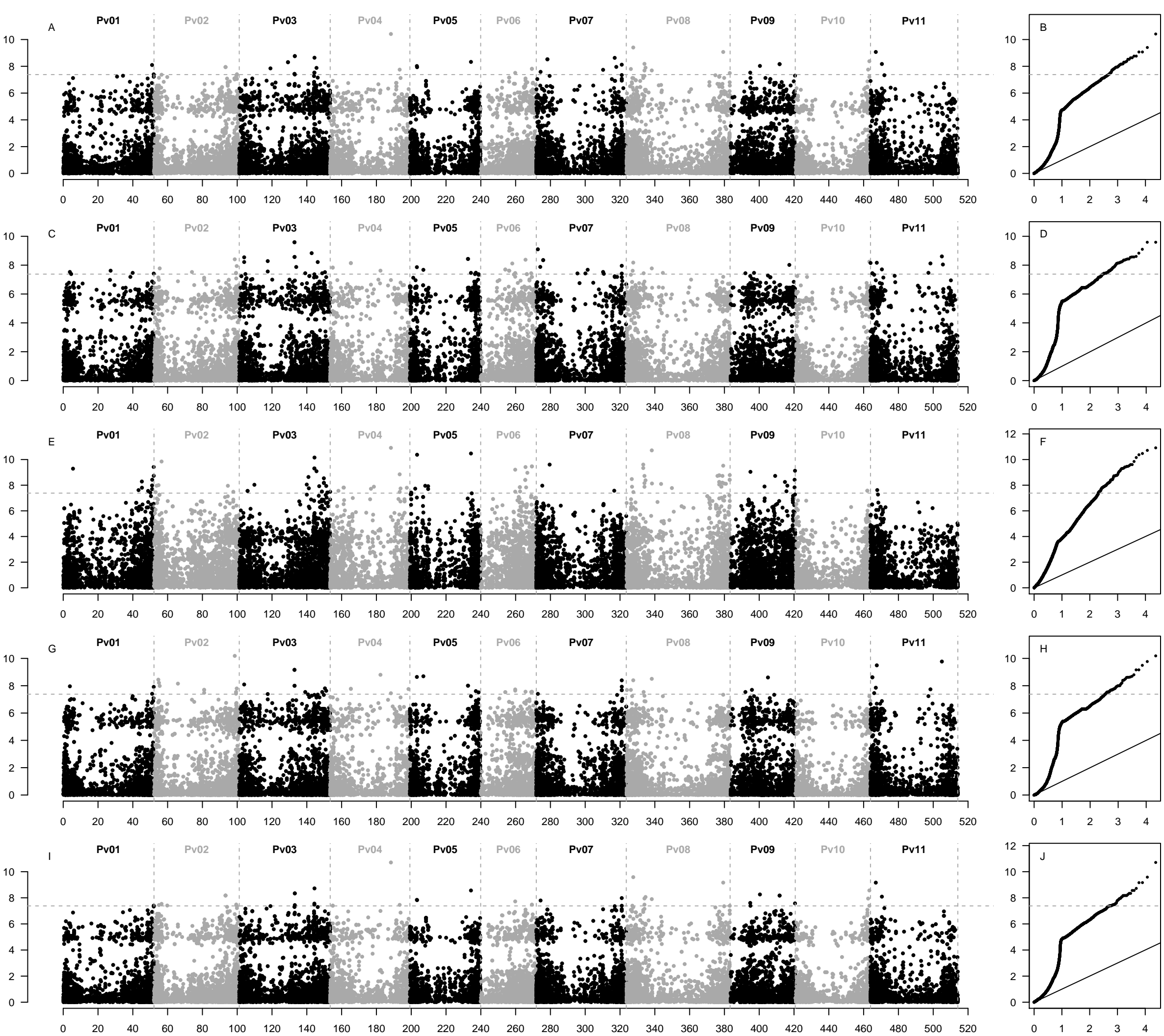

Supplement: FIGURE S3 — Genome–environment association (GEA) analyses for drought tolerance according to five different mixed linear models (MLMs) in 86 common bean accessions based on 22,845 GBS-derived SNP markers. Given are Manhattan plots (A,C,E,G,I) and QQ-plots (B,D,F,H,J) of –log10 (P-value) for the following models: (A,B) MLM with the genepool identity and the first two PCoA axes scores (Figure 2) as covariates; (C,D) MLM with the within-genepool subpopulation identity, according to Blair et al. (2012), and the first two PCoA axes scores (Figure 2) as covariates; (E,F) MLM with the first two PCoA axes scores (Figure 2) as covariates (model shown in Figure 3); (G,H) MLM with the within-genepool subpopulation identity, according to Blair et al. (2012), as covariate; and (I,J) MLM with the genepool identity as covariate. All models used a centered IBS kinship matrix as a random effect. The bioclimatic-based drought index follows Cortés et al. (2013). The gray dashed horizontal line marks the P-value threshold after Bonferroni-correction for multiple comparisons. Black and gray colors highlight different common bean (Pv) chromosomes. [file Image_3.pdf]
